# De Novo Atomistic Discovery of Disordered Mechanical Metamaterials by Machine Learning

**DOI:** 10.1002/advs.202304834

**Published:** 2024-01-25

**Authors:** Han Liu, Liantang Li, Zhenhua Wei, Morten M. Smedskjaer, Xiaoyu Rayne Zheng, Mathieu Bauchy

**Affiliations:** ^1^ SOlids inFormaTics AI‐Laboratory (SOFT‐AI‐Lab) College of Polymer Science and Engineering Sichuan University Chengdu 610065 China; ^2^ AIMSOLID Research Wuhan 430223 China; ^3^ Department of Ocean Science and Engineering Southern University of Science and Technology Shenzhen 518055 China; ^4^ Department of Chemistry and Bioscience Aalborg University Aalborg 9220 Denmark; ^5^ Department of Material Science and Engineering University of California Berkeley Berkeley CA 94720 USA; ^6^ Physics of Amorphous and Inorganic Solids Laboratory (PARISlab) Department of Civil and Environmental Engineering University of California Los Angeles CA 90095 USA

**Keywords:** Bayesian optimization, cellular materials, interparticle interactions, molecular dynamics simulation, stiffness‐density scaling

## Abstract

Architected materials design across orders of magnitude length scale intrigues exceptional mechanical responses nonexistent in their natural bulk state. However, the so‐termed mechanical metamaterials, when scaling bottom down to the atomistic or microparticle level, remain largely unexplored and conventionally fall out of their coarse‐resolution, ordered‐pattern design space. Here, combining high‐throughput molecular dynamics (MD) simulations and machine learning (ML) strategies, some intriguing atomistic families of disordered mechanical metamaterials are discovered, as fabricated by melt quenching and exemplified herein by lightweight‐yet‐stiff cellular materials featuring a theoretical limit of linear stiffness–density scaling, whose structural disorder—rather than order—is key to reduce the scaling exponent and is simply controlled by the bonding interactions and their directionality that enable flexible tunability experimentally. Importantly, a systematic navigation in the forcefield landscape reveals that, in‐between directional and non‐directional bonding such as covalent and ionic bonds, modest bond directionality is most likely to promotes disordered packing of polyhedral, stretching‐dominated structures responsible for the formation of metamaterials. This work pioneers a bottom‐down atomistic scheme to design mechanical metamaterials formatted disorderly, unlocking a largely untapped field in leveraging structural disorder in devising metamaterials atomistically and, potentially, generic to conventional upscaled designs.

## Graphical abstract

Through the integration of molecular dynamics simulations and machine learning, this work systematically investigates the role of melt‐quenched structural disorder in architecting mechanical metamaterials at the atomistic scale, leading to the atomistic discovery of disordered metamaterials, as exemplified by lightweight‐yet‐stiff cellular materials featuring a theoretical limit of linear stiffness–density scaling.

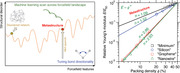

## 1 Introduction

Architected materials, an emerging family of structural materials, are constructed through the spatial combination of building blocks ranging in scale from (sub)microns to meters.^[^ ^1^, ^2^, ^3^ ^]^ This family constitutes a vast and flexible design space of mechanical properties that, by chance, exhibit beneficially unusual characteristics compared to their constitutive bulk elements.^[^ ^4^, ^5^, ^6^ ^]^ This phenomenon has led to the discovery of “mechanical metamaterials”, a term coined to differentiate them from their pristine bulk state and to highlight their impressive and distinctive mechanics.^[^ ^7^ ^]^ Despite their infinite architected tunability,^[^ ^4^, ^8^ ^]^ conventional metastructures are generally designed as ordered patterns to balance the structural simplicity and practical applicability.^[^ ^9^, ^10^ ^]^ In that regard, leveraging structural disorder in devising metamaterials would fully unlock their mechanical tunability, wherein, however, little is known about the role played by disordered metamaterials (if any).^[^ ^11^ ^]^

Unlike conventional ordered metamaterials, the architecture of disordered metamaterials requires more sophisticated and delicate fabrication rules for formatting structural disorder.^[^ ^6^, ^8^, ^12^, ^13^, ^14^, ^15^ ^]^ This area remains largely unexplored across the complexity spectrum of geometric disorder, considering both local irregularity and global non‐periodicity and hierarchy.^[^ ^15^, ^16^, ^17^ ^]^ While structural disorder can be induced through deliberate fabrication rules, finding a rule that allows systematic, flexible access to a wide spectrum of geometric disorder presents a grand challenge.^[^ ^8^ ^]^ In that regard, when scaling metastructures conceptually down to the atomistic or microparticle scale, the atomistic world inspires us with abundant disorder formats across different material families, microscopic interactions, and scales.^[^ ^18^, ^19^, ^20^ ^]^ Impressively, recent study inspires us with automatic design of topology‐accessible molecular assembly by devising its constitutive building blocks.^[^ ^21^ ^]^ However, built upon spatial arrangement of particle‐level blocks, such particle systems such as glasses are traditionally stereotyped as the pristine bulk elements of architected materials—rather than the architected material itself.^[^ ^22^, ^23^ ^]^ rendering it elusive whether mechanical metamaterials can be feasible at atomistic or microparticle level,^[^ ^6^, ^24^ ^]^ let alone the atomistic lesson of structural disorder in devising metamaterials.

**Figure 1:**
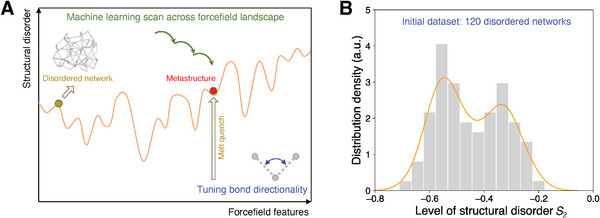
Diversifying structural disorder across forcefield landscape. A) Schematic of forcefield landscape, wherein the structural disorder exhibits a complex dependence on the forcefield features that dominate the formation of melt‐quenched structures. B) Distribution of the level of structural disorder in an initial dataset, which consists of 120 melt‐quenched structures prepared at different forcefield features. The level of disorder is quantified by two‐body excess entropy *S*_2_ (see Equation 1).

Here, through the integration of molecular dynamics (MD) simulations and machine learning (ML), we systematically investigate the role of structural disorder in architecting mechanical metamaterials at the atomistic scale. This approach leads to the atomistic discovery of disordered metamaterials, fabricated through melt‐quenching, as exemplified herein by lightweight‐yet‐stiff cellular materials featuring a theoretical limit of linear stiffness–density scaling.^[^ ^24^, ^25^ ^]^ Instead of directly manipulating structural modifications, we control structural disorder through the underlying forcefields that govern architected fabrications during melt quenching. This structural dependance on forcefield features creates a high‐dimensional forcefield landscape, as illustrated in **Figure** **1**. This allows for a machine learning‐based scan of the entire forcefield landscape (see Figure 1), inducing a diverse range of complexities in structural disorder to identify the optimal disordered state for forming metamaterials. Interestingly, contrary to conventional ordered metamaterials,^[^ ^9^, ^10^ ^]^ we observe that stiffness–density scaling tends to linearize with structural disorder at the atomistic scale, rendering it mechanically more robust to voids. A systematic exploration of the forcefield landscape reveals that, between directional and non‐directional bonding such as covalent and ionic bonds, modest bond directionality is most likely to promote the formation of disordered metamaterials. This is characterized by disordered polyhedral packing, which, upon loading, generically resembles conventional ordered metamaterials due to their bond stretching‐dominated nature.^[^ ^2^ ^]^ We expect these atomistic lessons would leverage structural disorder in devising metamaterials atomistically and, potentially, generic to conventional upscaled designs.

## 2 Results and Discussion

### 2.1 Diversifying Structural Disorder Across Forcefield Landscape

To establish our conclusions, we first fabricate atomistic networks in disordered patterns by globally manipulating the melt‐quenched structural disorder through their local forcefield. As part of their structural disorder, the architected networks exhibit some porous state whose porosity is tuned by their melt‐state packing density. Starting from a melt‐state packing density, the forcefield dominates the formation of a melt‐quenched structure and can be modified to tune its structural disorder.^[^ ^26^ ^]^ Figure 1A shows a schematic of forcefield landscape, wherein the structural disorder exhibits a complex dependence on the forcefield features. Here, we adopt an angular three‐body forcefield formulated by Stillinger–Weber (SW) potential to solely tune the bond directionality,^[^ ^27^ ^]^ including bonding angle and angular constraint strength. More forcefield details can be found in the Experimental Section.

Sampling from this forcefield landscape, we build an initial dataset consisting of 120 melt‐quenched structures at different forcefields. As the initial dataset incorporates all key features of different forcefield types (see Section S1, Supporting Information), the initial dataset can modestly represent the topography of the forcefield landscape (see Section S2, Supporting Information). Taking the example of one melt‐state packing density at 20%, Figure 1B shows the distribution of the level of structural disorder in the initial dataset, where the level of disorder is quantified by two‐body excess entropy *S*_2_, as described in the following:^[^ ^28^, ^29^ ^]^

(1) $$S_{2}=\frac{k_{B}\rho }{2}\;\int \left[g\left(r\right)-\left(g\left(r\right)-1\right)\right]dr$$

where 𝜌 is the particle number density, *k*_B_ = 1 is the Boltzmann constant in reduced unit, *g*(*r*) is the pair distribution function, and the cutoff is set as 2.2 herein to cover the first two or three coordination shells. Notably, by tuning bond directionality, the resultant disordered networks exhibit a wide spectrum of disorder level, ranging from *S*_2_ = −0.2 to −0.7, where *S*_2_ = 0 refers to the maximum disordered limit, and a crystalline network generally lies around *S*_2_ ≈ −1. Overall, these results demonstrate the extensive capacity of the forcefield approach in diversifying structural disorder.

**Figure 2:**
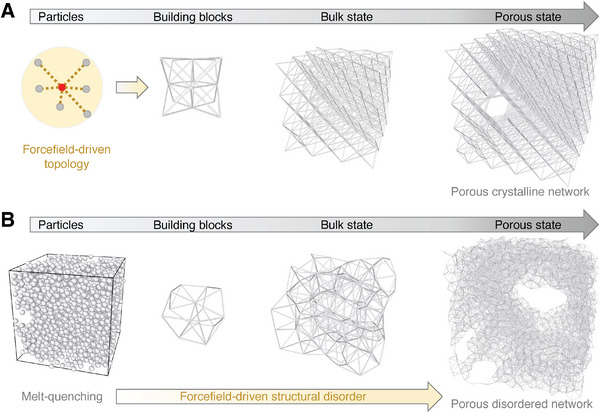
Illustration of architecting disordered metamaterials at atomistic scale. A) Fabrication of a porous crystalline network in analogy to conventional ordered metamaterials. The ordered topology is locally equilibrated by its forcefield, and the pores are randomly introduced into the network to mimic the formation of imperfect crystal.^[^ ^24^, ^30^ ^]^ B) Fabrication of a porous disordered network in analogy to its crystalline counterpart by melt quenching. The structural disorder including the porous state is globally governed by its local forcefield.

**Figure** **2** illustrates the architected fabrication of disordered networks by melt quenching, in analogy to conventional upscaled architected materials built upon spatial combination of building blocks,^[^ ^3^ ^]^ which is generally formatted as ordered patterns to simplify their structural complexity and, by scaling bottom down, their atomistic counterparts, i.e., the crystalline networks (see Figure 2A)—are also fabricated to compare with their disordered analogy, where the porous state is randomly introduced to mimic the formation of imperfect crystals.^[^ ^24^, ^30^ ^]^ Note that both the ordered and disordered networks are structurally controlled by their forcefield landscape in terms of both the local topology and the global metastability.^[^ ^26^, ^31^, ^32^ ^]^ More details of the forcefield and the fabrication of porous networks are described in the Experimental Section.

### 2.2 Linearizing Stiffness–Density Scaling by Structural Disorder

**Figure 3:**
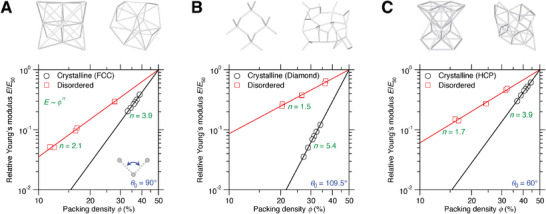
Linearizing stiffness–density scaling by structural disorder. The scaling exponent *n* is compared between disordered porous networks and their crystalline counterparts, including A) FCC‐type, B) diamond‐type, and C) HCP‐type, where the energy‐favored bond angle 𝜃_0_ = 90°, 109.5°, and 60°, respectively, and the Young's modulus *E* is normalized by their extrapolated value *E*_50_ at the packing density Φ = 50% to highlight the stiffness–density slope comparison in logarithmic scale.

We now investigate the stiffness–density scaling of porous networks in both the crystalline and disordered formats. **Figure** **3** shows the comparison of Young's modulus *E* as a function of packing density Φ between crystalline and disordered state in the same forcefield landscape of Face‐Centered Cubic (FCC)‐type, diamond‐type, and Hexagonal Closest Packed (HCP)‐type, respectively (see **Table** **1** for forcefield parameters). Indeed, we find that, as commonly known in cellular materials,^[^ ^33^, ^34^, ^35^ ^]^ the Young's modulus *E* exhibits a power law dependance on the packing density Φ, that is,

(2) $$\frac{E}{E_{\mathrm{ref}}}={\left(\frac{\phi }{\phi_{\mathrm{ref}}}\right)}^{n}\;$$

where *n* is the stiffness–density scaling exponent, and the subscript “ref” refers to a reference state that is herein selected as the extrapolated state at Φ = 50% for the crystalline and disordered networks, respectively, so as to normalize the stiffness–density scaling and to highlight the comparison of stiffness–density slope *n* between crystalline and disordered networks in logarithmic scale.

**Table 1:** Parameter sets of all forcefields labeled in this work. Two‐body forcefield parameters are fixed based on ref. [27] while three‐body forcefield parameters are variable. Reduced unit is used for all quantities.

Forcefield labels	Two‐body parameters							Three‐body parameters			Scaling exponent *n*
	𝜖	𝜎	*a*	*A*	*B*	*p*	*q*	𝜆/*A*	𝛾	𝜃_0_ [°]	
FCC	1.0	1.0	1.8	7.0495	0.6022	4.0	0.0	4.000	1.250	90.0	3.90 (Crystalline) 2.07 (Disordered)
Diamond/Silicon								3.000	1.200	109.5	5.44 (Crystalline) 1.52 (Disordered)
HCP								1.000	1.250	60.0	3.90 (Crystalline) 1.74 (Disordered)
Disordered graphene								3.000	0.800	120.0	2.86
Disordered nanowire								3.000	1.200	180.0	2.93
Global minimum								2.094	1.206	109.7	1.09
Covalent								4.0	1.206	109.7	1.63
Ionic								0.0	1.206	109.7	1.22
Competitive minimum 1								3.645	1.383	89.1	1.22
Competitive minimum 2								3.246	1.588	118.0	1.23
Competitive minimum 3								2.100	1.604	179.3	1.22

Obviously, the scaling exponent *n* is a structure‐dominant parameter, and smaller *n* leads to less stiffness loss upon density decreasing, potentially beneficial to make lightweight‐yet‐stiff metamaterials.^[^ ^36^, ^37^, ^38^ ^]^ It is interesting that, unlike conventional upscaled metamaterials whose magnitude of *n* is controlled by their structural order of building blocks,^[^ ^39^, ^40^ ^]^ the structural disorder tends to linearize the stiffness–density scaling in the atomistic world more than their crystalline counterparts in a generic and undemanding fashion (see Figure 3). This result highlights the largely untapped opportunity of leveraging structural disorder to fabricate disordered metamaterials, which are likely mechanically more robust to voids than their crystalline counterparts.

**Figure 4:**
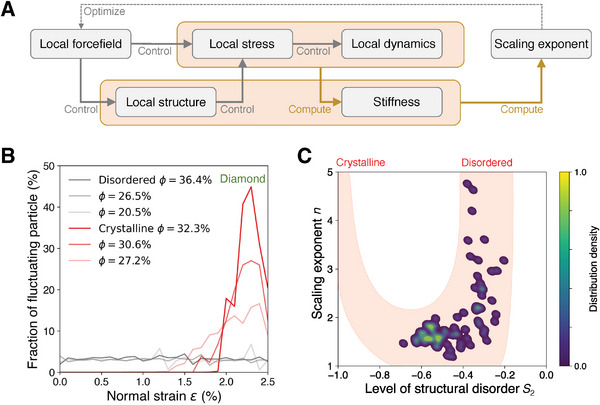
Correlation between structural disorder and stiffness–density‐scaling exponent. A) Chain of reasoning from local forcefield to scaling exponent. B) Fraction of fluctuating particle as a function of normal strain for diamond‐type disordered versus crystalline network upon tensile deformation. The fluctuating particle is detected by TimeSOAP approach.^[^ ^41^, ^42^ ^]^ C) Distribution density of scaling exponent n as a function of the level of structural disorder *S*_2_ in the initial dataset. The shadow region delineates a trend to guide the eyes, where the *S*_2_ values of crystalline networks are located ≈−1.3.

To unveil the correlation between structural disorder and the linearized scaling, **Figure** **4**A provides a chain of reasoning from forcefield landscape to the resultant stiffness–density scaling, wherein local forcefield determines the scaling exponent through i) manipulating interparticle interaction and ii) dominating the melt‐quenched structure. Upon axial loading, the force experienced by each particle is controlled by the local spatial arrangement of neighbor particles. As the force drives particle dynamics iteratively, the system evolves to exhibit strain and stress for stiffness computation.^[^ ^20^ ^]^ Finally, the structure and stiffness pairs are built to assess the stiffness–density scaling exponent. When tracing back this reasoning chain, local forcefield is the sole origin to trigger the chain activation and responsible for its resultant scaling exponent *n*. As such, the scaling exponent can be minimized by solely optimizing its prior forcefield.

According to the reasoning chain (see Figure 4A), the different exponent between disordered and crystalline network can be ascribed to a significant difference of local deviatoric stress responsible for highly fluctuating particles (see Section S3, Supporting Information), thus leading to distinct local dynamics. Figure 4B shows the fraction of fluctuating particle as a function of normal strain for disordered versus crystalline porous networks upon tensile deformation, where the fluctuating particle is detected by TimeSOAP approach,^[^ ^41^, ^42^ ^]^ that is, a new method exceling at detecting highly fluctuating particles from a time series of configuration evolution (see Section S4, Supporting Information). Interestingly, by reducing packing density, disordered network is mechanically robust to voids and remains a low level of <5% fluctuating defects contributing to the stiffness loss. In contrast, by introducing voids to crystalline network, the onset of fluctuating defects occurs early and quickly surges to a high fraction level of >10%, thus dramatically lowering the stiffness. Overall, these results demonstrate that structural disorder has a tendency to linearize stiffness–density scaling through i) suppressing the fraction surge of fluctuating particles upon elastic deformation and ii) restricting the total fraction of fluctuating particles upon decreasing packing density.

Finally, Figure 4C provides the distribution density of scaling exponent *n* as a function of the level of structural disorder *S*_2_ in the initial dataset. It is interesting to see a U‐shape trend of exponent *n* with respect to the disorder level *S*_2_. Note that crystalline networks have a lower‐bound *S*_2_ ≈ −1 but exhibit large scaling exponent *n*. When approaching the upper‐bound limit *S*_2_ = 0, disordered networks exhibit a large variation in scaling exponent *n*, wherein the high exponent *n* is likely ascribed to the high‐*S*_2_ structures formed by loose packing (see Section S2, Supporting Information). Therefore, it is the region in‐between the lower and upper *S*_2_ bound most likely to offer a minimal exponent *n*. These results necessitate machine learning exploration to optimize the structural disorder toward minimum scaling exponent.

### 2.3 Optimizing Structural Disorder in Their Forcefield Landscape by Machine Learning

**Figure 5:**
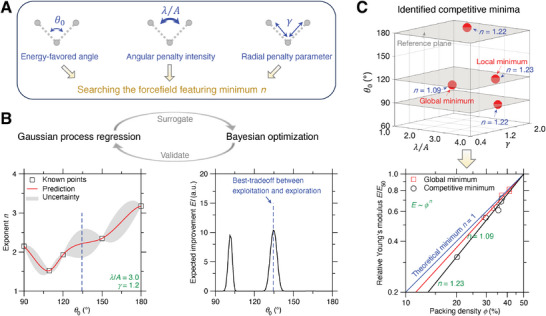
Searching the forcefield featuring linear stiffness–density scaling by machine learning (ML). (A) Illustration of the forcefield parameter space. The three‐body interaction in reduced units is tuned by energy‐favored angle 𝜃_0_, angular penalty intensity 𝜆/A, and radial penalty parameter 𝛾. (B) ML searching scheme using Gaussian process regression (GPR) and Bayesian optimization (BO).^[^ ^43^, ^44^ ^]^ Based on an initial dataset (square points), GPR model offers the prediction (red line) of scaling exponent *n* and its uncertainty (grey area) as a function of the forcefield parameters.^[^ ^45^ ^]^ Utilizing the GPR prediction and its uncertainty, the BO model predicts the next forcefield candidate that offers the highest expected improvement (EI) best balancing the exploitation of GPR prediction and the exploration of its uncertainty area.^[^ ^46^ ^]^ (C) ML‐identified competitive minima of stiffness–density scaling exponent n in the forcefield parameter space, with their stiffness–density slope approaching the theoretical limit of linear scaling in logarithmic scale.^[^ ^25^, ^47^ ^]^

Although the stiffness–density scaling exponent *n* is largely reduced by its structural disorder, it remains unknown what types of structural disorder is optimal in reducing *n* and architecting the disordered metamaterials thereof. Here, based on machine learning (ML), we explore the entire forcefield landscape that governs the structural disorder ranging over an extensive design space, so as to identify the optimal structural disorder minimizing the scaling exponent *n*. **Figure** **5**A illustrates the tunable space of an archetypal forcefield formulated by three‐body interaction that imposes angular constraints to the central atom,^[^ ^27^ ^]^ where the radial two‐body interaction is fixed in reduced units to provide a generic reference for the role played by angular three‐body constraints, which is herein tuned by the energy‐favored angle 𝜃_0_, angular penalty intensity 𝜆/*A*, and radial penalty parameter 𝛾. More details of the forcefield are described in the Experimental Section.

Figure 5B shows the ML strategy in searching the forcefield featuring minimum scaling exponent *n* by integrating Gaussian process regression (GPR) and Bayesian optimization (BO).^[^ ^43^, ^44^ ^]^ Starting from an initial dataset of forcefield‐and‐exponent pairs, the GPR model offers not only a prediction but also its uncertainty for the entire forcefield parameter space.^[^ ^45^ ^]^ Utilizing the GPR prediction and its uncertainty, the BO model predicts the next forcefield candidate that offers the highest expected improvement (EI),^[^ ^46^ ^]^ which provides a best balance between the exploitation of GPR prediction and the exploration of its uncertainty area—considering that the minimum is most likely located in either the minimum regions of GPR prediction or the maximum regions of GPR uncertainty. By iteratively updating the dataset of forcefield‐and‐exponent pairs via high‐throughput MD simulations, the integration strategy of GPR and BO holds the promise to identify the optimal forcefield featuring minimum *n* in the forcefield parameter space. More details of the ML strategy can be found in the Experimental Section. Based on the optimization scheme that systematically explores the forcefield landscape, several forcefields (see Table 1 for forcefield parameters) have been discovered to induce structural disorder patterns that are featured by the competitive minima *n*, as illustrated in Figure 5C, which approach the theoretical minimum of linear stiffness–density scaling,^[^ ^25^, ^47^ ^]^ i.e., *n* ≈ 1, promising to fabricate lightweight‐yet‐ultrastiff disordered metamaterials.

Note that, although the melt‐quenching simulation is reduced to 0 K to filter out the kinetic energy contribution from stiffness computation, the melt‐quenched structures have reached their metastable state by energy minimization and remain valid at finite temperature. If the system temperature is elevated to finite temperature, the particle dynamics would be gradually activated by increasing temperature. Although these structures are destined to lose stability at extremely high finite temperature, they generally exhibit low or modest relaxation behavior below the temperature away from their glass transition temperature or fictive temperature *T*_f_.^[^ ^48^ ^]^ Herein, *T*_f_ is generally above 0.03 in reduced unit, which is equivalent to 755 K in the silicon energy scale of SW potential^[^ ^27^ ^]^ (see Section S5, Supporting Information).

Despite the structural stability present herein, we must pay attention to different molecular design rules that may significantly affect the stability of their final structures, such as a molecular design strategy proposed recently to explore the energy‐stable molecular topology by tuning bond angles of building blocks and assembling the building blocks into different types of topological cages.^[^ ^21^ ^]^ Theoretically, it is true that some configurations are more unstable than others and occupy higher energy state in their potential energy landscape (PEL).^[^ ^26^, ^31^ ^]^ From the PEL viewpoint, the structural stability of our melt‐quenched structures is likely originated from the fact that, by tuning forcefield parameters, the topography of PEL varies accordingly but our melt‐quenching rule enables each structure to fully relax in its own PEL with no confinement, so as to reach a deep local minimum. As such, this approach can offer structural design exhibiting satisfactory stability and is translatable for different types of molecular design.

Besides the structural stability, it is worth pointing out different forcefield characteristics are likely to influence the stiffness‐to‐density scaling, including short‐ versus long‐range interaction, three‐ versus many‐body interaction, bond directionality, asymmetry, etc. Although this work focuses on the influence of short‐range bonding directionality on stiffness‐to‐density scaling, it deserves deliberate investigation of the other forcefield characteristics that influence the scaling behavior. Note that the SW potential is a simplified version of interatomic forcefield dedicated to describing covalent system or angular three‐body interactions. This simplification is both a strength and weakness: it facilitates more efficient optimization of forcefield parameters but, as a compromise, the simulation accuracy may not be guaranteed.^[^ ^27^, ^49^ ^]^ We have demonstrated that SW potential can accurately simulate disordered covalent systems consisting of short‐range radial and angular interactions (see Section S7, Supporting Information).

Moreover, instead of blindly unlocking the variability of all parameters in SW potential, we intentionally select the key parameters that tune bond directionality, i.e., angle value and angle strength. This allows us to filter out and keep away from any unrealistic regions of SW potential, considering that both the angular value and strength parameters show no dependance or impose no influence on any other parameters of SW potential. In other words, the SW potential excels at tuning bond directionality in terms of angular value and strength, which are two general basic attributes widely existing in covalent‐bonding systems or angular‐constrained systems, regardless of strong or weak angular interaction. Therefore, by tuning bond directionality in SW potential, the structural phenomena found in this work reveal the general basic attributes of any realistic covalent‐bonding systems or angular‐constrained systems.

### 2.4 Unveiling the Structural and Forcefield Features in Disordered Metamaterials

**Figure 6:**
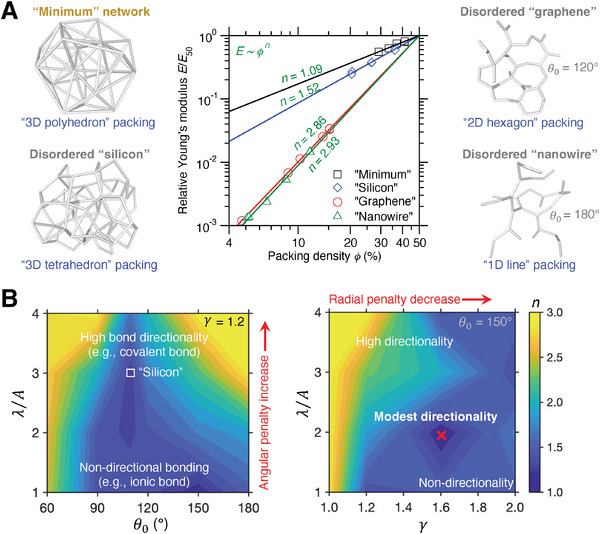
Structural and forcefield features in disordered metamaterials exhibiting linear stiffness–density scaling. A) Comparison of the local structures between the metamaterial (“minimum” network) and other typical disordered networks, including “silicon”‐type, “graphene”‐type, and “nanowire”‐type, where the four networks are architected by random packing of polyhedrons, tetrahedrons, hexagons, and lines, respectively. Their stiffness–density slopes in logarithmic scale are also provided for comparison. B) Topography of the stiffness–density scaling exponent n in some selected sections of forcefield parameter space, where the two selected planes are 𝛾 = 1.2 and 𝜃_0_ = 150°, respectively, and the disordered “silicon” network (square point) is located in the 𝛾 = 1.2 plane. The red marker indicates the minimum position.

We now take a closer inspection into the structural and forcefield features in the ML‐identified disordered metamaterials exhibiting a nearly linear stiffness–density scaling, denoted by “minimum” network. **Figure** **6**A provides a comparison of local structures between the minimum network and other typical disordered networks, including “silicon”‐, “graphene”‐, and “nanowire”‐type networks (see Table 1 for forcefield parameters). These archetypal structures are prepared by the same melt‐quenching simulation protocol as the minimum network but imposing different forcefields listed in Table 1, respectively, in agreement with their distinct bond directionality features. Details of the simulation protocol are described in the Experimental Section. Notably, the four types of networks are architected by random packing of polyhedrons, tetrahedrons, hexagons, and lines, respectively, where the 3D polyhedral or tetrahedral blocks lead to disordered packings featuring relatively small stiffness–density scaling exponent and, in contrast, much larger exponents are obtained by spatial packing of 2D hexagonal or 1D linear blocks, in agreement with the previous finding of 3D graphene‐assembly.^[^ ^50^ ^]^ Indeed, compared to lower‐dimensional building blocks, 3D blocks are more likely resistant to deformation upon loading (see below), in agreement with the previous finding of nanoporous self‐assembled silicas.^[^ ^51^ ^]^ These results highlight polyhedral packing as a pivotal structural feature to fabricate disordered metamaterials with mechanically robust 3D networks.

Besides the structural features, we further investigate the forcefield features in disordered metamaterials by a systematic navigation of stiffness–density scaling in the forcefield landscape. Figure 6B shows the topography of the scaling exponent *n* in two anatomized planes of the forcefield parameter space, i.e., the 𝛾 = 1.2 plane and 𝜃_0_ = 150° plane, where the disordered “silicon”‐type network and a competitive “minimum” network are located, respectively. On the one hand, by increasing the angular and radial penalty in the angular constraints, the local forcefield exhibits high bond directionality similar to the covalent bonding,^[^ ^27^ ^]^ leading to relatively high scaling exponent *n* that is likely ascribed to the enhanced barrier of spatial polyhedral packing by highly directional bonding. On the other hand, the local forcefield with little angular constraints results in non‐directional bonding, as exemplified by ionic or metallic bond,^[^ ^20^, ^26^ ^]^ which is beneficial to the formation of polyhedral networks but tends to be susceptible to deformation via structural reorganization upon loading without bond directionality.^[^ ^52^, ^53^, ^54^ ^]^ As such, we find that, in‐between directional and non‐directional bonding, modest bond directionality (e.g., ionocovalent bond^[^ ^32^ ^]^) is most likely to minimize the scaling exponent *n* by forming mechanically robust polyhedral networks resistant to deformation when subjected to loads. Overall, these results reveal the key role of polyhedral networks with modest bond directionality in fabricating disordered metamaterials.

**Figure 7:**
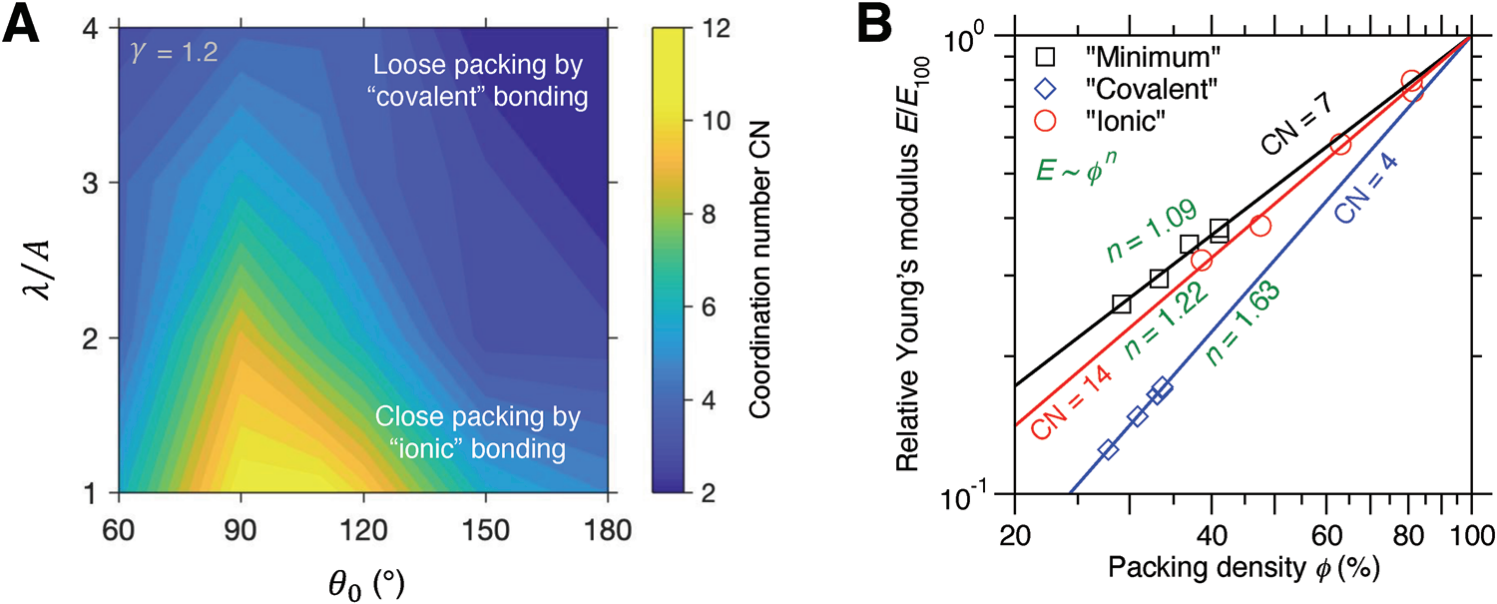
Compensation effect between local packing density and bond directionality. A) Topography of maximum‐likelihood coordination number (CN) as a function of forcefield parameter at the 𝛾 = 1.2 plane. B) Comparison of stiffness–density scaling for the “minimum,” “covalent,” and “ionic” network, where the “ionic” and “covalent” structures are relaxed from the “minimum” network.

Next, based on the chain of reasoning (see Figure 4A), we investigate the influence of bonding types on the scaling exponent from their bond directionality and local structure formation. **Figure** **7**A shows the maximum‐likelihood coordination number (CN) in a disordered network as a function of forcefield parameter. As expected, by tuning the angular strength, the nondirectional ionic‐like bonding and directional covalent‐like bonding lead to close and loose packing, respectively. Moreover, when the bonding angle exceeds 120°, the local structure tends to become loose‐packed open structure. Notably, these low‐ and high‐CN regions largely overlap with, respectively, the high‐ and low‐exponent regions in the forcefield space (see Figure 6B). This result echoes the fact that compared to high‐CN structure, the low‐CN structure is more likely to promote deviatoric deformation and facilitate the onset of fluctuating particles, resulting in more pronounced stiffness loss. However, the packing structure is not solely responsible for the scaling exponent. Upon closer inspection, there exists some mismatch between the CN and exponent topography, wherein some low‐CN regions such as disordered silicon can exhibit local minimum exponent than its surrounding higher‐CN regions. This mismatch indicates somehow the contribution of certain “compensating” interparticle interactions that govern particle dynamics, if tracing back the reasoning chain (see Figure 4A).

To reveal this compensating forcefield effect, we generate both an “ionic” and a “covalent” structure relaxed from the “minimum” network, with the angular penalty intensity 𝜆/*A* = 0.0 and 4.0, respectively (see Table 1). The simulation details are described in the Experimental Section. Figure 7B shows the stiffness–density scaling for the three structures, wherein the structures have been relaxed to their metastable state but exhibit very different local packing structure, with CN = 7, 14, 4 for minimum, ionic, and covalent network, respectively. Interestingly, compared to the minimum network, the ionic network is highly close‐packed structure with twice CN to resist deviatoric deformation and, despite its competitive small exponent *n*, the absence of angular constraints makes the local structure prone to reorganize under deviatoric stress, leading to a slightly larger exponent *n*. Similarly, compared to the ionic network, the covalent network is a very loose‐packed structure susceptible to deviatoric deformation but, as compensation, the strong angular constraints contribute to prevent local shear and the onset of fluctuating particles. Overall, these results demonstrate a compensation effect between local packing density and bond directionality in determining stiffness–density scaling of melt‐quenched structures.

### 2.5 Bridging Disordered to Ordered Metamaterials by Bond Stretching‐Dominated Nature

Finally, we investigate the bond stretching versus bending response in the disordered metamaterials when subjected to loads, in comparison with conventional ordered metamaterials built on bond stretching‐dominated structures.^[^ ^2^, ^14^, ^55^ ^]^ To characterize the bond stretching versus bending response, we compute, respectively, the shift of normal and shear stress per atom with respect to zero strain upon loading,^[^ ^56^ ^]^ where the normal and shear stress per atom are thermodynamically ill‐defined without the concept of ensemble statistics but, from a practical perspective, enable the quantification of bond‐stretching and bond‐bending responses, respectively.^[^ ^18^ ^]^ The computation methodology of stress shift per atom is described in the Experimental Section. This local stress state influences exponent *n* in two ways: First, the normal stress shift per atom directly promotes the normal stress and stiffness thereof, while the shear stress shift per atom has very limited contribution. Second, the shear stress shift per atom promotes local deviatoric deformation that causes the onset of fluctuating particles and the stiffness loss thereof^[^ ^18^, ^48^, ^54^ ^]^ (see Section S3, Supporting Information). From this perspective, structure disorder can linearize stiffness–density scaling through i) more bond‐stretching response that promote normal stress shift per atom and ii) less bond‐bending response that harmfully facilitate shear stress shift per atom.

**Figure 8:**
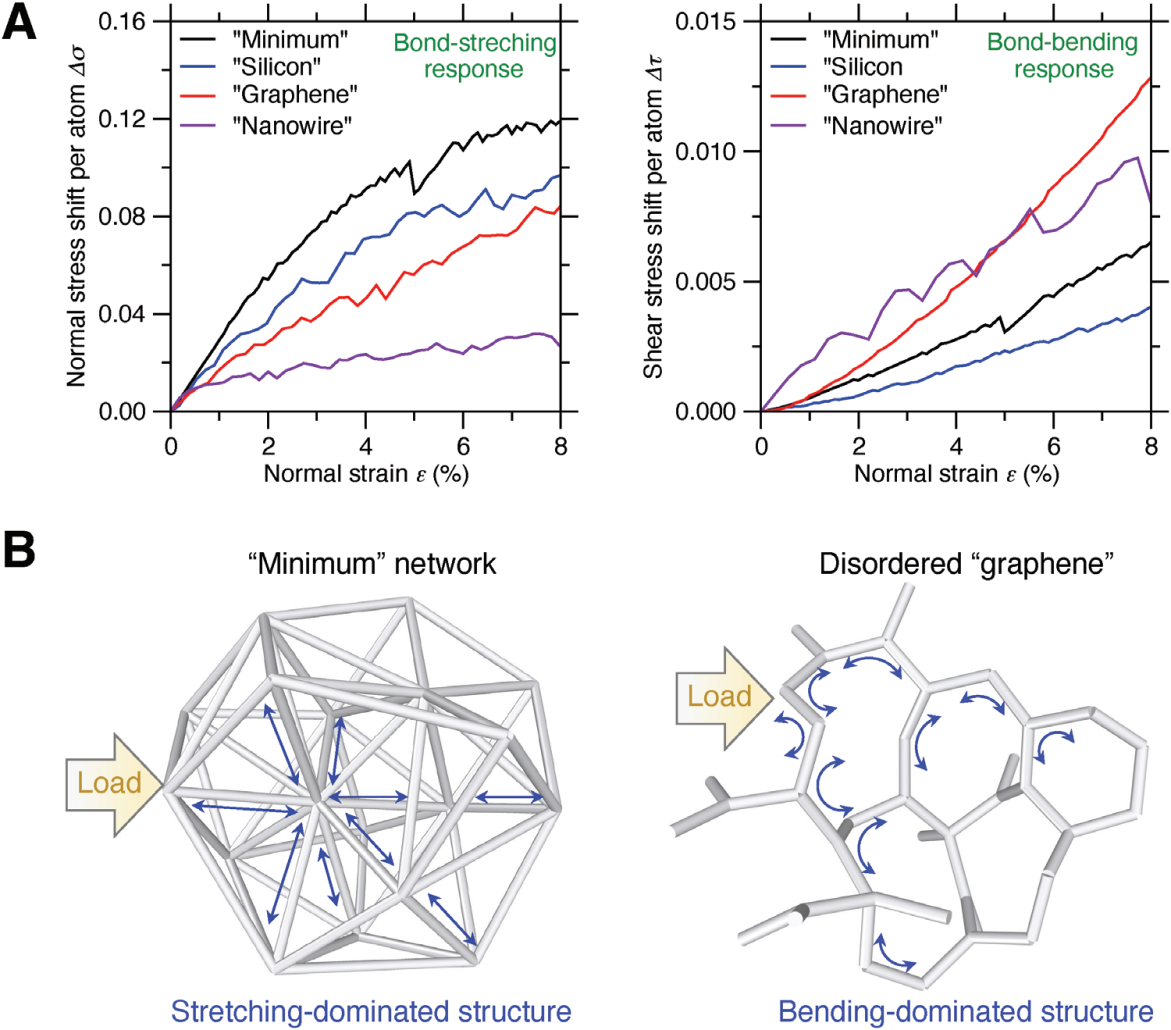
Bond stretching versus bending response in disordered metamaterials upon loading. A) Evolution of the normal (left panel) versus shear (right panel) stress shift per atom in the metamaterial (“minimum” network), with respect to its normal strain when subjected to uniaxial deformation, and the result of disordered “silicon,” “graphene,” and “nanowire” are added for comparison. B) Illustration of the bond stretching‐dominated versus relatively bending‐dominated response in, respectively, the “minimum” and “graphene” network when subjected to loading.

**Figure** **8**A shows the normal and shear stress shift per atom as a function of the normal strain of “minimum” network when subjected to uniaxial deformation, and the result of disordered “silicon,” “graphene,” and “nanowire” are added for comparison. Indeed, we find that, compared to the graphene‐type or nanowire‐type network, the minimum or silicon‐type network exhibits more pronounced bond‐stretching response but relatively negligible bond‐bending behavior, as illustrated in Figure 8B, while the hexagonal blocks of graphene network are more susceptible to bond bending upon loading. Regarding the 3D structure comparison between minimum and silicon network, we find that the minimum network offers both higher normal and shear stress shift per atom. Although shear stress shift may harmfully cause the onset of fluctuating particle, the higher coordination number of minimum network makes it more resilient to local deviatoric deformation upon shear, thus compensating the risk of fluctuating particles. These results confirm the stretching‐dominated nature of disordered metastructures, in the same spirit of conventional ordered metamaterials,^[^ ^2^, ^14^ ^]^ thus unifying the ordered and disordered metamaterials within a universal architected principle, that is, small and large stiffness‐density scaling exponent *n* are governed by stretching‐ and bending‐dominated structures, respectively.

## 3 Conclusion

Together, this work conceptually translates the architected materials to atomistic scale and discovers some atomistic families of disordered mechanical metamaterials, as exemplified by linearizing the stiffness–density scaling. Unlike conventional upscaled metamaterials built on ordered patterns, the disordered metamaterials leverage structural disorder in devising architected materials that are potentially mechanically more robust to voids. Importantly, inspired by the abundant disorder formats of atomistic systems, the melt‐quenched fabrication rule of formatting structural disorder is delivered to enable a flexible tunability across a wide spectrum of geometric disorder, by systematically scanning the entire forcefield landscape that governs the abundant formats of structural disorder. Interestingly, we find that, in‐between directional and non‐directional bonding such as covalent and ionic bonds, modest bond directionality is most likely to induce polyhedral, stretching‐dominated structures that are beneficial to the formation of disordered metamaterials. We envision that these atomistic lessons would unlock new opportunities of leveraging structural disorder in devising mechanical metamaterials atomistically and, potentially, generic to conventional upscaled designs.

## 4 Experimental Section

### Description of Three‐Body Interaction by Stillinger–Weber Potential

The forcefield is formulated as a three‐body interaction to regulate the bond directionality and is described by Stillinger–Weber potential that is well‐established for tetrahedral silicon network,^[^ ^27^ ^]^ i.e., a combination of (i) radial two‐body interaction *U*_2_ and (ii) angular three‐body interaction *U*_3_ to compute the interatomic potential energy *U* between a central atom *i* and its two neighbor atoms *j* and *k*:

(3) $$U\;\left(r_{ij},r_{ik},\theta_{ijk}\right)=\sum_{i}\;\sum_{j>i}U_{2}\left(r_{ij}\right)+\sum_{i}\sum_{j\neq i}\sum_{k>j}U_{3}\left(r_{ij},r_{ik},\theta_{ijk}\right)$$

where *U* is a function of the interatomic distance *r_ij_, r_ik_*, and the bond angle 𝜃_ijk_ between the vectors *r_ij_* and *r_ik_*. The explicit formulations of *U*_2_ and *U*_3_ are provided in the following:^[^ ^27^ ^]^

(4) $$U_{2}\;\left(r_{ij}\right)=A_{ij}\;\varepsilon_{ij}\left[B_{ij}{\left(\frac{\sigma_{ij}}{r_{ij}}\right)}^{p_{ij}}-{\left(\frac{\sigma_{ij}}{r_{ij}}\right)}^{q_{ij}}\right]\exp\left(\frac{\sigma_{ij}}{r_{ij}-a_{ij}\sigma_{ij}}\right)$$

(5) $$\begin{array}{ccc} U_{3}\;\left(r_{ij},r_{ik},\theta_{ijk}\;\right) & = & \lambda_{ijk}\;\varepsilon_{ijk}{\left(\cos\theta_{ijk}-\cos\theta_{0ijk}\right)}^{2}\exp\left(\frac{\gamma_{ij}\sigma_{ij}}{r_{ij}-a_{ij}\sigma_{ij}}\right)\\ & & \exp\left(\frac{\gamma_{ik}\sigma_{ik}}{r_{ik}-a_{ik}\sigma_{ik}}\right) \end{array}$$

where 𝜖 and 𝜎 defines the bond energy and length scale, respectively, 𝜆/*A* yields the relative angular penalty intensity with respect to its two‐body interaction magnitude, 𝜃_0_ is the energy‐favored angle, 𝛾 is a radial penalty parameter in the angular term, the forcefield cutoff is set as *a*𝜎, and all other parameters in the two‐body term were determined based on ref. [27] to keep the minimum bond energy equal to 𝜖 at the equilibrium bond length (i.e., 1.12𝜎 for silicon^[^ ^27^ ^]^). Here, the parameters were kept in two‐body energy term *U*_2_ fixed to investigate the effect of angular three‐body constraint term *U*_3_, and all quantities adopt reduced units to make this study generic across different materials families and scales. Table 1 provides the parameter sets of all forcefields labeled in this work.

### Fabrication of Porous Networks by MD Simulations

The porous disordered networks were prepared by melt quenching MD simulations. The initial configure was prepared by randomly placing atoms into a cubic box of side length *L* = 40 with periodic boundary condition, and an energy minimization step was applied to prevent atomic overlaps.^[^ ^57^ ^]^ The initial packing density was set as 20%, 26%, and 40% to tune the final porosity, where the packing density was computed as the atomic volume fraction with an effective atomic diameter equal to the equilibrium bond length 1.12𝜎.^[^ ^18^, ^27^ ^]^ All simulations were conducted under *NPT* ensemble using LAMMPS package,^[^ ^58^ ^]^ and the timestep was fixed as 0.01. Starting from the initial configuration, the system first undergoes a melt simulation at high temperature *T* = 0.3 for a duration of 100,^[^ ^59^ ^]^ where the system pressure was gradually reduced to zero from its initial pressure if positive or half of its initial pressure if negative—note that the positive and negative sign of pressure indicate system under compression and tension in LAMMPS convention, respectively,^[^ ^56^, ^58^ ^]^ and the barostat pressure was a key parameter to tune the final porosity. Then the system temperature was subsequently decreased from 0.3 to 0 temperature to freeze the structural disorder within duration of 100, where the pressure is kept zero. Finally, an energy minimization step is applied to mimic the annealing process that removes the internal stress.^[^ ^57^ ^]^

In the same spirit, the porous crystalline network was prepared by first creating a crystalline lattice of 10 × 10 × 10 unit cells using the equilibrium bond length 1.12𝜎,^[^ ^27^ ^]^ with periodic boundary condition applied to the box. Then, mimicking the formation of imperfect crystals,^[^ ^24^, ^30^ ^]^ the atoms were randomly removed in the box to a prescribed porosity (e.g., removing 5%, 10%, 15%, 20% of all atoms), followed by an energy minimization step to stabilize the configuration.^[^ ^57^ ^]^ Starting from the initial configuration, all remaining simulations were the same as that for porous disordered networks, except that the initial melt simulation was replaced by a relaxation simulation at a relatively modest temperature *T* = 0.03 to promote atom mobility without being melt.^[^ ^59^ ^]^ Note that, at low porosity of removing <20% atoms, the stabilized configurations remain crystalline state, while much higher porosity would lead to disordered configurations. Finally, a relaxation simulation of the “minimum” network was conducted to generate the “ionic” and “covalent” network. Using the “minimum” network as initial configuration, the same relaxation protocol was adopted as that for crystalline networks, so that the structures were fully relaxed to their metastable state under the “ionic” and “covalent” forcefield (see Table 1), respectively.

### Assessment of Stiffness–Density Scaling Exponent at Each Forcefield

After fabricating a set of porous networks with various packing density under the same forcefield, the stiffness tensor was then computed for each configuration by subjecting the simulation box to a series of axial and shear plane deformations along each Cartesian axis,^[^ ^18^ ^]^ where each deformation increment is set as 0.05%, followed by an energy minimization step,^[^ ^57^ ^]^ and the maximum strain for each deformation was restricted to ±0.5%. The corresponding changes in the system's potential energy ∂*U*_s_ and strain ∂*e*_𝛼_ defines six stress components *s*_α_:^[^ ^18^ ^]^

(6) $$s_{\alpha }=\frac{1}{V}\;\frac{\partial U_{s}}{\partial e_{\alpha }}$$

and 36 elastic constants *C*_𝛼𝛽_:^[^ ^18^ ^]^

(7) $$C_{\alpha \beta }=\frac{1}{V}\;\frac{\partial^{2}U_{s}}{\partial e_{\alpha }\partial e_{\beta }}$$

where 𝛼 and 𝛽 are the Cartesian direction indexes. All configurations were found to be nearly fully isotropic. The Young's modulus *E* was then calculated from the stiffness tensor.^[^ ^60^ ^]^ Finally, the stiffness–density scaling exponent *n* was obtained by a linear fit between Young's modulus and packing density in logarithmic scale following Equation (2).

### Machine Learning Using Gaussian Process Regression and Bayesian Optimization

Finally, by integrating the simulation‐based assessment module into a machine learning (ML) pipeline, the ML model would accelerate the navigation in the forcefield landscape toward minimum scaling exponent. Here, the ML strategy combines Gaussian process regression (GPR) and Bayesian optimization (BO).^[^ ^43^, ^44^ ^]^ First, to interpolate the forcefield landscape by GPR, first an initial dataset of forcefield parameter sets was established, their corresponding scaling exponents were computed by the simulation‐based assessment module. The forcefield parameters of the initial dataset were selected as an orthogonal array that contains all combinations from the energy‐favored 𝜃_0_ = 60°, 90°, 109.5°, 120°, 150°, and 180°, the angular penalty intensity 𝜆/*A* = 1.0, 2.0, 3.0, and 4.0, and the radial penalty parameter 𝛾 = 0.4, 0.8, 1.2, 1.6, and 2.0 (see Section S1, Supporting Information). Based on the initial dataset, the GPR model offers a prediction of scaling exponent and its uncertainty for each point in the forcefield parameter space, by correlating the point at prediction with all known points in the space via a multivariate Gaussian distribution formulation,^[^ ^45^ ^]^ enabling us to estimate a Gaussian‐type probability distribution of the point at prediction. Details about the GPR formulation can be found in refs. [43, 45].

Then, relying on an acquisition function—i.e., expected improvement EI(*x*) herein^[^ ^43^, ^46^ ^]^—that utilizes the GPR prediction *n*(*x*) and its uncertainty ∆*n*(*x*), the BO model acts as a surrogate model to determine the next optimal forcefield to try, that is,

(8) $$\mathrm{EI}\;\left(x\right)=\left\{\begin{array}{cc} \left[n\left(x_{\min}\right)-n\left(x\right)\right]D_{c}\left(Z\right)+\Delta n\left(x\right)D_{p}\left(Z\right) & \mathrm{if}\;\Delta n\left(x\right)>0\\ 0 & \mathrm{if}\;\Delta n\left(x\right)=0 \end{array}\right.\;$$

where *x* is a point in the forcefield parameter space, *n*(*x*_min_) denotes the current minimum scaling exponent *n* in the dataset, *D*_c_(*Z*) and *D*_p_(*Z*) are the cumulative distribution and probability density function of the standard normal distribution, respectively. By construction, the value of EI(*x*) is high (i) when the expected value *n*(*x*) is higher than the *n*(*x*_min_) or (ii) when the uncertainty ∆*n*(*x*) is high.^[^ ^43^, ^46^ ^]^ This EI function behavior is beneficial if considering the fact that the global minimum position is most likely located in (i) either the region near the present minimum position or (ii) the region with very high uncertainty. Therefore, it is natural to expect that the EI function keeps tiny value in all areas but reaches a summit at the position both near present minimum and highly uncertain, and by construction, the EI function is capable of working in this way in an efficient and accurate fashion. As such, the candidate forcefields with the highest EI values offer a best balance between (i) the exploitation that minimizes the scaling exponent *n*(*x*) and (ii) the exploration that minimizes the uncertainty ∆*n*(*x*).^[^ ^43^, ^46^ ^]^ By iteratively updating the dataset with these informative datapoints, the ML strategy provides an efficient navigation to the optimal forcefield.

### Computation of Normal and Shear Stress Shift Per Atom

Finally, the bond stretching and bending response of a structure was analyzed upon loading by computing the normal and shear stress shift per atom. Although stress per atom is thermodynamically ill‐defined without the concept of ensemble statistics, this computation expresses the individual contribution of each particle *i* to the virial of the system, convenient to capture the local stress state *P_i_* applied to particle *i*:^[^ ^18^, ^56^ ^]^

(9) $$P_{i}=\frac{m_{i}v_{i}^{2}+{\vec{r}}_{i}\cdot {\vec{F}}_{i}}{3V_{i}}\;$$

where *P_i_* consists of three normal stress components {$\sigma_{i}^{x}$, $\sigma_{i}^{y}$, $\sigma_{i}^{z}$} along x, y, z axis and three shear stress components {$\tau_{i}^{xy}$, $\tau_{i}^{yz}$, $\tau_{i}^{xz}$} in xy, yz, and xz plane, respectively, *V_i_*, *m_i_*, *v_i_*, and ${\vec{r}}_{i}$ are the volume, mass, velocity, and position of the particle *i*, respectively, and ${\vec{F}}_{i}$ is the resultant of the force applied on the particle *i* by all the other particles in the system. Here, the volume *V_i_* of each particle was defined based on its Voronoi volume. Note that, although the network as a whole was at zero pressure, some bonds are under compression while others are under tension, so that they mutually compensate each other. By convention, a positive normal stress represents here a state of tension, whereas a negative one represents a state of compression.

The normal stress per atom $\bar{\sigma }$ was computed by averaging the three normal stress components 𝜎*_i_* for atom *i* and, subsequently, averaging over all atoms in the system. Similarly, the shear stress per atom $\bar{\tau }$ is calculated by computing the von Mises definition of shear stress τ*_i_* for atom *i* and, subsequently, averaging over all atoms in the system, as formulated below: ^[^ ^61^ ^]^

(10) $$\bar{\sigma }=\langle \sigma_{i}\rangle \;=\langle \frac{\sigma_{i}^{x}+\sigma_{i}^{y}+\sigma_{i}^{z}}{3}\;\rangle$$

(11) $$\bar{\tau }=\langle \tau_{i}\rangle \;=\langle \frac{\sqrt[{2}]{{\left(\tau_{i}^{xy}\right)}^{2}+{\left(\tau_{i}^{yz}\right)}^{2}+{\left(\tau_{i}^{xz}\right)}^{2}}}{3}\rangle$$

where < > is an average operation over all atoms in the system. When the system was subjected to uniaxial tensile deformation, the {$\bar{\sigma }$, $\bar{\tau }$} pair was recorded at each normal strain and, by taking the zero‐strain configuration as the reference, the normal and shear stress shift was computed per atom 𝛥𝜎 and 𝛥𝜏 from the subtraction between the current state of {$\bar{\sigma }$, $\bar{\tau }$} and its initial state. This approach allows to characterize the tendency of local volumetric and deviatoric deformation, as linked to bond stretching and bending response, respectively.

## Conflict of Interest

The authors declare no conflict of interest.

## Author Contributions

H.L. and M.B. contributed to conceptualization and methodology of this article. H.L. and L.L. performed investigation and visualization. Supervision was done by H.L., Z.W., M.S., X.Z. and M.B. H.L. wrote the original draft. H.L., L.L., Z.W., M.S., X.Z., and M.B. reviewed and edited the final manuscript.

## Supporting information

Supporting Information

## Data Availability

The data that support the findings of this study are available from the corresponding author upon reasonable request.
