# Supplementary material for: De Novo Atomistic Discovery of Disordered Mechanical Metamaterials by Machine Learning
Source: Adv Sci (Weinh). 2024 Jan 25;11(13):2304834. doi: 10.1002/advs.202304834 (PMC10987143; doi:10.1002/advs.202304834)
Supplement: Supplementary file 1 — Supporting Information [file ADVS-11-2304834-s001.pdf]

ADVANCED SCIENCE
Open Access

Supporting Information

for *Adv. Sci.*, DOI 10.1002/advs.202304834

De Novo Atomistic Discovery of Disordered Mechanical Metamaterials by Machine Learning

Han Liu, Liantang Li, Zhenhua Wei, Morten M. Smedskjaer, Xiaoyu Rayne Zheng and Mathieu Bauchy**

Supplementary Materials to "De Novo Atomistic Discovery of Disordered Mechanical Metamaterials by Machine Learning"

Han Liu [a,b] *, Liantang Li [a,b], Zhenhua Wei [c], Morten M. Smedskjær [d], Xiaoyu Rayne Zheng [e], Mathieu Bauchy [f] *

[a] *SOlids inFormaTics AI-Laboratory (SOFT-AI-Lab), College of Polymer Science and Engineering, Sichuan University, Chengdu 610065, China*

[b] *AIMSOLID Research, Wuhan 430223, China*

[c] *Department of Ocean Science and Engineering, Southern University of Science and Technology, Shenzhen 518055, China*

[d] *Department of Chemistry and Bioscience, Aalborg University, Aalborg 9220, Denmark*

[e] *Department of Material Science and Engineering, University of California Berkeley, Berkeley, CA, 94720 USA*

[f] *Physics of AmoRphous and Inorganic Solids Laboratory (PARISlab), Department of Civil and Environmental Engineering, University of California, Los Angeles, California, 90095, USA*

* *Corresponding author: Han Liu (happylife@ucla.edu), Mathieu Bauchy (bauchy@ucla.edu)*

Contents

S1. Initial dataset of forcefield parameters 2

S2. Forcefield effect on two-body excess entropy 2

S3. Stress shift per atom in disordered versus crystalline porous network 3

S4. Fraction of fluctuating particle detected by TimeSOAP approach 4

S5. Stability of atomistic configurations at finite temperature 8

S6. Simulation reliability of SW potential 9

References 11

S1. Initial dataset of forcefield parameters

The initial forcefield parameters constitute a 3D matrix of mesh-grid coordinates from the three forcefield variables θ_0 = {60°, 90°, 109.5°, 120°, 150°, 180°}, λ/A = {1.0, 2.0, 3.0, 4.0}, and γ = {0.4, 0.8, 1.2, 1.6, 2.0}, as illustrated in Fig. S1. As this 3D grid matrix incorporates all key features of different forcefield types, the initial dataset can modestly represent the topography of the forcefield landscape.

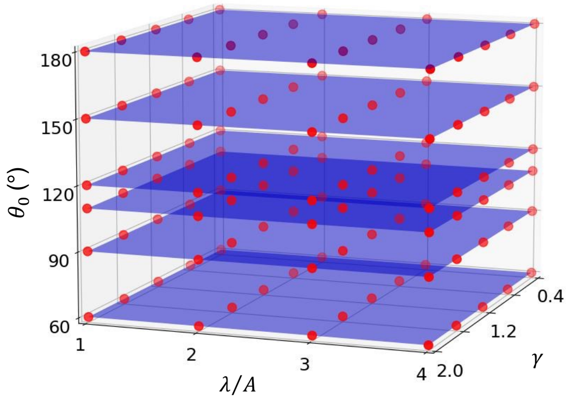

Fig. S1: Initial dataset of forcefield parameters. The red points represent the forcefields selected to construct the initial dataset. The shadow planes are to guide the eyes.

S2. Forcefield effect on two-body excess entropy

Figure S2 shows the topography of two-body excess entropy S_2 in selected planes of the forcefield parameter space. Indeed, S_2 shows a complex dependance on the forcefield parameters. Upon closer inspection, the S_2 value generally declines with respect to the increase of bond directionality, in accordance with the loose packing of directional covalent-type bonding (see Fig. 7A in the main text). Interestingly, we find that the S_2 topography shows a diagonal symmetry at the slice plane of γ = 1.2, where larger bond angle is more likely to yield high-S_2 structure, suggesting the formation of some loose-packed open structures when large bond angle is energy-favored.

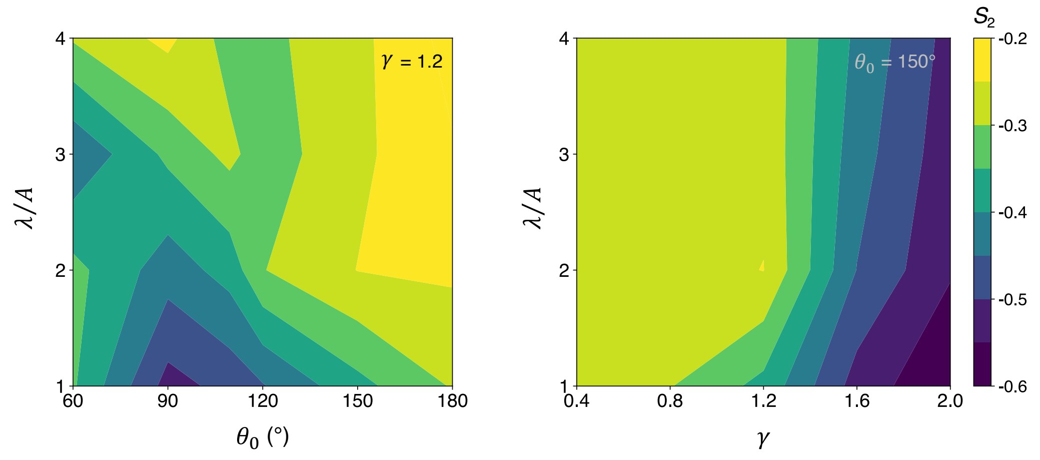

Fig. S2: Topography of two-body excess entropy S_2 as a function of forcefield parameters at γ = 1.2 (left) and θ_0 = 150° (right) plane.

S3. Stress shift per atom in disordered versus crystalline porous network

Taking the "diamond" forcefield as an example, its crystalline and disordered network share the same forcefield but exhibit a pronounced difference in exponent *n*. According to the reasoning chain (see Fig. 4A in the main text), the different exponent can be ascribed to a significant difference of local stress state in disordered versus crystalline network, thus leading to distinct local dynamics (see Fig. 4B in the main text). Figure S3 provides the normal and shear stress shift per atom as a function of normal strain in disordered versus crystalline network upon tensile deformation. Notably, the disordered network exhibits higher normal stress shift but much lower shear stress per atom than its crystalline counterpart.

This local stress state influences exponent *n* in two ways: Firstly, the normal stress shift per atom directly promotes the normal stress and stiffness thereof, while the shear stress shift per atom has very limited contribution. Second, the shear stress shift per atom promotes local deviatoric deformation that causes the onset of fluctuating particles and the stiffness loss thereof. From this perspective, structure disorder can

linearize stiffness–density scaling through (i) more bond-stretching response that promote normal stress shift per atom and (ii) less bond-bending response that harmfully facilitate shear stress shift per atom.

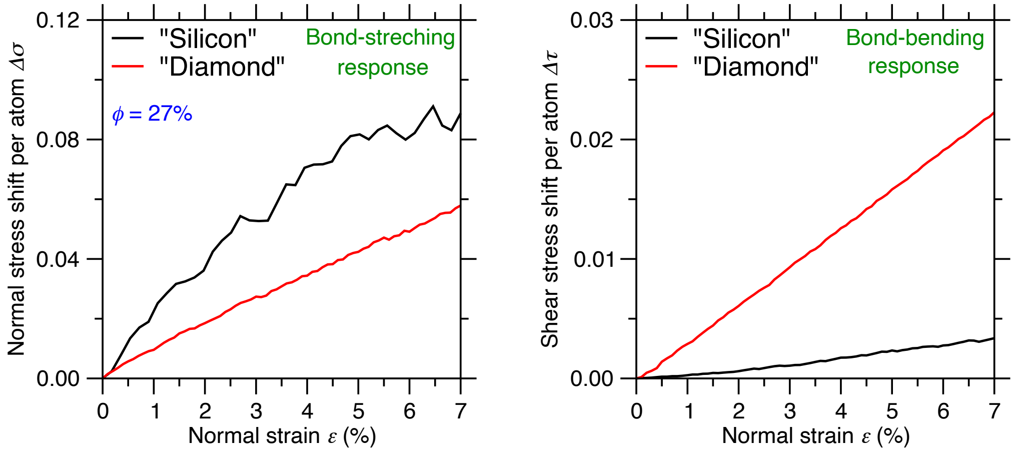

Fig. S3: Comparison of normal (left) and shear (right) stress shift per atom as a function of normal strain in silicon-type disordered versus crystalline porous network upon uniaxial tensile deformation. Both structures exhibit similar packing density ϕ = 27%.

S4. Fraction of fluctuating particle detected by TimeSOAP approach

Here, we conduct a TimeSOAP analysis to unveil the connection between structural disorder and the observed linearization in stiffness–density scaling (see Fig. 3 in the main text). The TimeSOAP approach excels at detecting the highly fluctuating particles from a time series of configuration evolution [[1,2]]. Following this approach, we first compute the vector of SOAP many-body descriptors $q_{\text{soap}}(i)$ for the local neighborhood environment of particle i, that is, the normalized form of partial power spectrum vector p_i [[3,4]], namely,

$$q_{\text{soap}}(i) = p_i / \sqrt{p_i \cdot p_i} \qquad \text{Eq. (S1)}$$

where the vector p_i consists of a set of elements $\{p_{nn'l}(i)\}$ defined in the following [[3,4]]:

$$p_{nn'l}(i) = \pi\sqrt{\frac{8}{2l+1}}\sum_m c_{nlm}(i)c_{n'lm}(i) \qquad \text{Eq. (S2)}$$

where n and n' are indices for the different radial basis function $g_n(r)$ up to $n_{\max}$ (herein, $n_{\max}$=8 and $g_n(r)$ adopts spherical gaussian type orbitals), and l is the angular degree of the spherical harmonics $Y_{lm}(\theta, \phi)$ up to $l_{\max}$ (herein, $l_{\max} = 6$).

The coefficient c_{nlm} is defined as the following inner product [[3,4]]:

$$c_{nlm}(i) = \iiint_{R^3} dV g_n(r)Y_{lm}(\theta,\phi)\rho(i,r) \qquad \text{Eq. (S3)}$$

where R^3 denotes the integral sphere centered at particle i with a cutoff radius R_{cut} (herein, $R_{\text{cut}} = 2.2$), and $\rho(i, r)$ is the gaussian smoothed particle density at a distance r for all particles j within the cutoff sphere [[3,4]]:

$$\rho(i,r) = \sum_{j\in R^3} \exp\left(\frac{-(r-r_{ij})^2}{2\sigma^2}\right) \qquad \text{Eq. (S4)}$$

where r_{ij} is the distance between the center particle i and its neighbor particle j. Here, we implement the SOAP calculation using the Dscribe package[5], and the output SOAP many-body descriptor vector $q_{\text{soap}}(i)$ contains 252 normalized elements $\{p_{nn'l}\}$ for each particle i. The environment similarity between local packing of particle i and j is measured by the SOAP distance $d_{\text{soap}}(i, j)$ [[3,4]]:

$$d_{\text{soap}}(i,j) = \sqrt[2]{2-2q_{\text{soap}}(i)q_{\text{soap}}(j)} \qquad \text{Eq. (S5)}$$

where $d_{\text{soap}}(i, j)$ ranges from 0 to 1 that represent, respectively, identical or zero-overlapping local environment between atom i and j.

Based on the SOAP distance, we calculate the TimeSOAP value $\lambda_i^{\varepsilon+\Delta\varepsilon}$ of particle i at the normal strain ε by [[1,2]]:

$$\lambda_i^{\varepsilon+\Delta\varepsilon} = d_{\text{soap}}(\varepsilon,\varepsilon+\Delta\varepsilon)/\Delta\varepsilon \qquad \text{Eq. (S6)}$$

where the SOAP distance d_{soap} is calculated between two normal strain states ε and $\varepsilon + \Delta\varepsilon$ for the same particle i, and the normal strain increment $\Delta\varepsilon$ is set as 0.1%. This TimeSOAP quantity captures the degree of dissimilarity between two local packing structures at normal strain ε and $\varepsilon + \Delta\varepsilon$, respectively. Figure S4 shows the evolution of TimeSOAP signals with respect to normal strain for all particles in the silicon-type disordered and crystalline porous network upon uniaxial tensile deformation, wherein the TimeSOAP values are normalized by multiplying 0.1%. At each normal strain, we find that some particles exhibit higher TimeSOAP value than others, and crystalline network shows very low TimeSOAP value in the initial deformation but quickly increases afterwards. By decreasing the packing density, we find that the patterns of TimeSOAP signals remain similar for disordered networks, while the patterns of crystalline networks show distinct difference, with lower packing density resulting in high-TimeSOAP particles at smaller normal strain.

To further characterize the particle dynamics, we compute the first derivative of TimeSOAP signal $\dot{\lambda}_i^{\varepsilon+\Delta\varepsilon}$ for each particle i at the normal strain ε by [[1,2]]:

$$\dot{\lambda}_i^{\varepsilon+\Delta\varepsilon} = \frac{\lambda_i^{\varepsilon+\Delta\varepsilon} - \lambda_i^{\varepsilon}}{\Delta\varepsilon} \qquad \text{Eq. (S7)}$$

which captures the rate of local environment change for each particle i during tensile deformation. Figure S5 shows the first derivative of TimeSOAP signals for silicon-type disordered versus crystalline porous network at different packing densities, wherein the derivative values are normalized by multiplying 0.1%. Indeed, the disordered network has a fraction of highly fluctuating particles at each normal strain, while particles in the crystalline network remains affine deformation with negligible structural fluctuation at initial strains. By decreasing the packing density, crystalline network shows earlier onset of fluctuating particles, while disordered network has more or less similar signal derivative patterns.

Here, we quantify the fraction of fluctuating particles at each strain by counting the number of particles that exhibit signal derivative values larger than 0.01, that is, a criterion determined as the position between two peaks in the distribution density of signal derivative values (see Fig. S5). The main text has discussed the

fraction evolution of fluctuating particles with respect to normal strain in the different systems (see Fig. 4B in the main text).

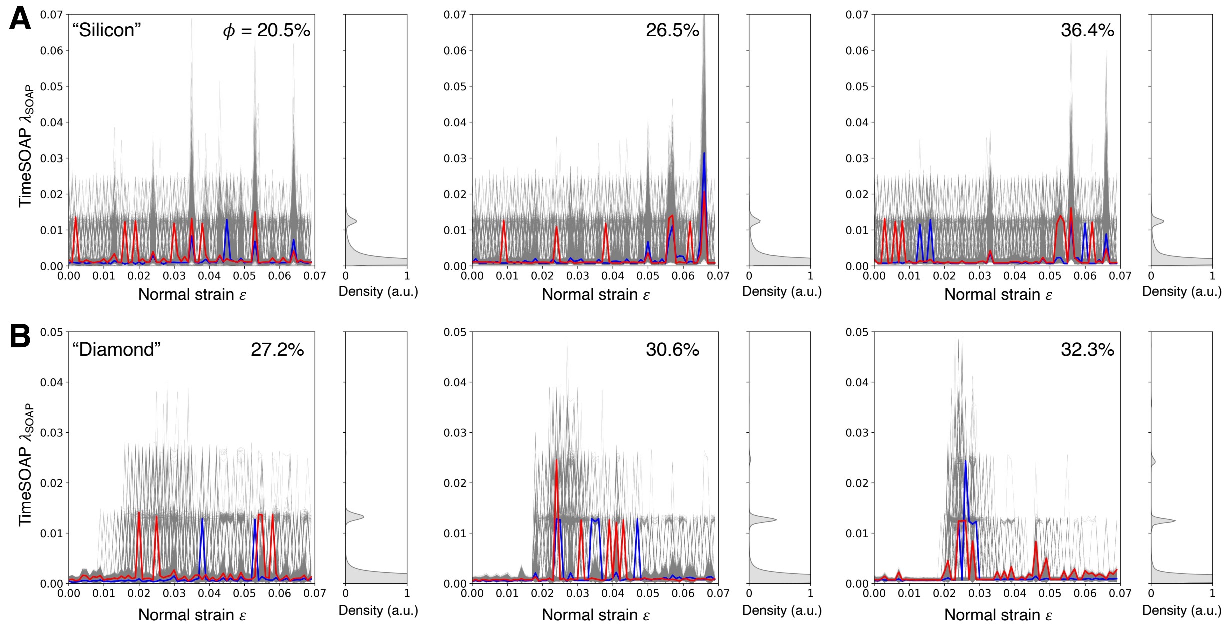

Fig. S4: TimeSOAP signals of (A) silicon-type disordered versus (B) crystalline porous network upon uniaxial tensile deformation. The grey lines are TimeSOAP signals for all particles in the system. The distribution density of signal values is computed in the right. The red and blue lines are highlighted to provide the typical signal examples. For both disordered and crystalline network, three packing densities are selected to exemplify the TimeSOAP analysis.

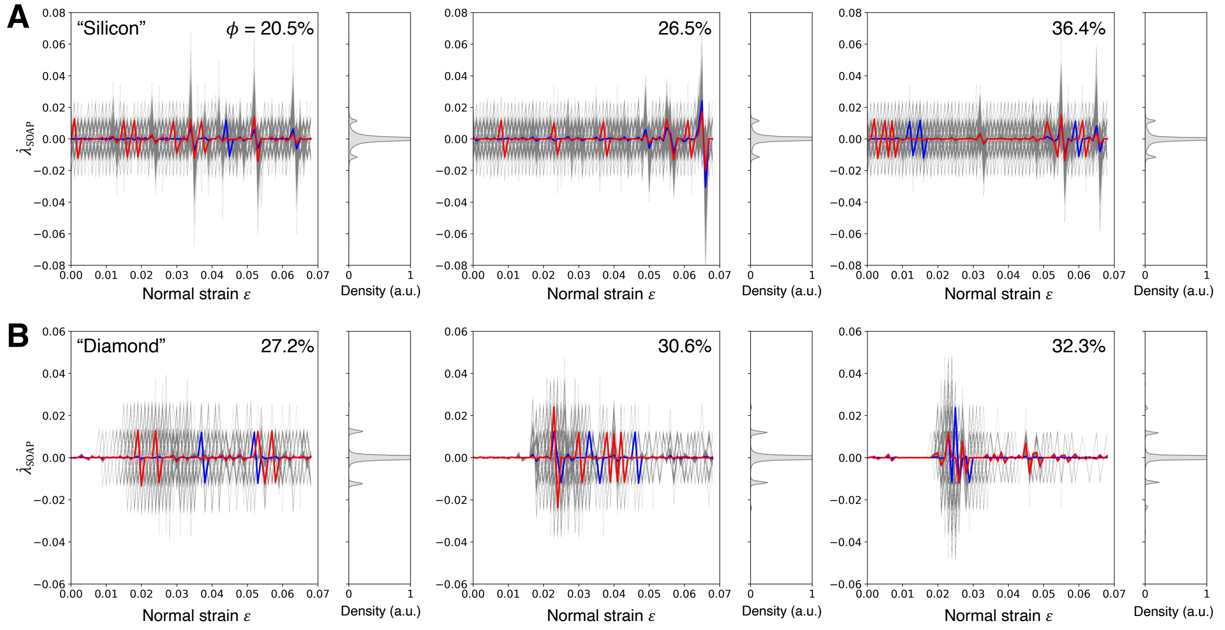

Fig. S5: First derivative of TimeSOAP signals for (A) silicon-type disordered versus (B) crystalline porous network at different packing densities. The distribution density of signal derivative values is computed in the right.

S5. Stability of atomistic configurations at finite temperature

We examine herein the structural stability of different typical structures present in this work. Figure S6 shows the fictive temperature of different structures and their particles' mean square displacement (MSD) with time at various temperatures below T_f. The simulation settings are described in the main text. We find that T_f is generally above 0.03 in reduced unit, which is equivalent to 755 K in the silicon energy scale of SW potential [[6]]. When the system temperature T = 0.03, we find that the particle MSD in different structures enter into their diffusion regime (or subdiffusive regime) but remain a very confined level within ~1 particle diameter displacement after a long simulation time of 1000 in reduced unit, equivalent to ~100 picoseconds in the silicon time scale of SW potential. When the system temperature T < 0.03, the particles

in different structures generally show very limited mobility through confined vibration motions around their initial positions. These results demonstrate the extensive stability of different structures presents in this work at modest finite temperature.

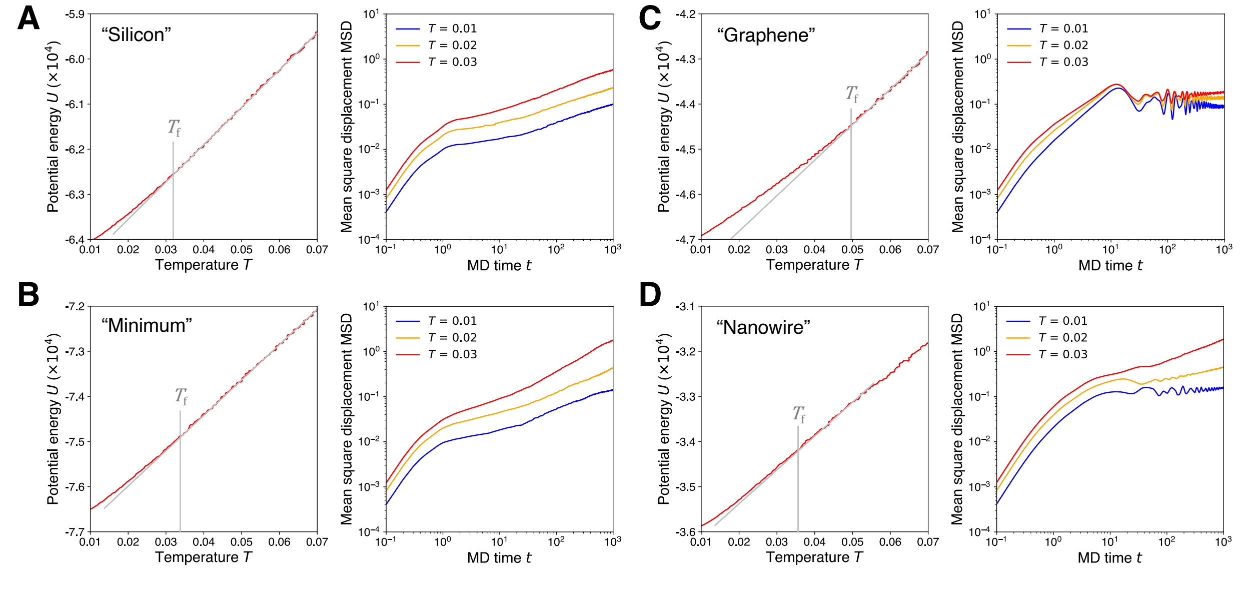

Fig. S6: Structural stability of (A) silicon-type, (B) minimum, (C) graphene-type, and (D) nanowire-type network. The left panel shows the potential energy as a function of system temperature during melt quenching, where the vertical line indicates the fictive temperature T_f. The right panel shows the particle mean square displacement as a function of MD time in logarithmic scale at system temperature $T = 0.01$, 0.02, and 0.03, respectively.

S6. Simulation reliability of SW potential

To validate the simulation reliability of SW potential, we could examine two extreme disordered networks, i.e., a network with no angular constraint and a strong covalent silicon network. The former network is solely described by the two-body interaction term of SW potential [[6]], that is, a generalized format of

Lennard–Jones potential suitable for describing short-range two-body interaction. By adding strong angular interaction term of SW potential, the disordered silicon exhibits tetrahedral packing structure similar to that prepared by an accurate EDIP potential as a reference—i.e., a well-established forcefield to describe disordered silicon phase [[7]], and their stiffness–density scaling comparison is provided in Fig. S7. Note that both the SW and EIDP potential adopts real units of silicon attributes for fair comparison. The timestep is fixed to 1 fs. The melt quenching simulation is the same as the reduced-unit simulation described in the main text, except that the melt-state temperature is set as 5000 K. We find that the two forcefields offer very close scaling results, suggesting that the SW potential is capable of simulating disordered silicon and, generically, other similar covalent systems consisting of short-range radial and angular interactions.

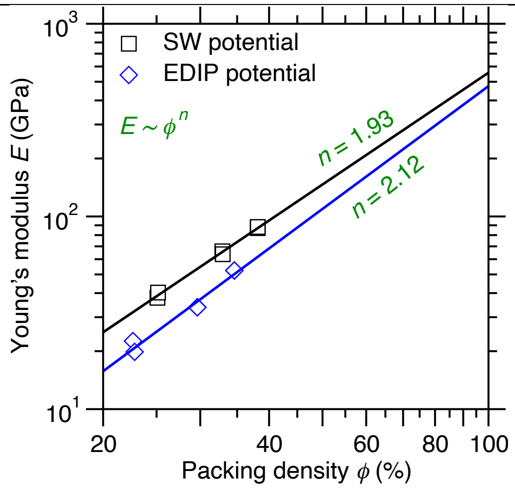

Fig. S7: Comparison of stiffness–density scaling for disordered silicon network generated by SW [[6]] and EDIP [[7]] potential.

Based on the simulation reliability of the two extreme scenarios, we then intentionally select the key parameters that tune bond directionality, i.e., angle value and angle strength, instead of blindly unlocking the variability of all parameters in SW potential. This allows us to filter out and keep away from any unrealistic regions of SW potential, considering that both the angular value and strength parameters show no dependance or impose no influence on any other parameters of SW potential. In other words, the SW

potential excels at tuning bond directionality in terms of angular value and strength, which are two general basic attributes widely existing in covalent-bonding systems or angular-constrained systems, regardless of strong or weak angular interaction. Therefore, by tuning bond directionality in SW potential, the structural phenomena found in this work reveal the general basic attributes of any realistic covalent-bonding systems or angular-constrained systems.

References

[1] C. Caruso, A. Cardellini, M. Crippa, D. Rapetti, G. M. Pavan, *The Journal of Chemical Physics* **2023**, *158*, 214302.

[2] M. Crippa, A. Cardellini, C. Caruso, G. M. Pavan, *Proceedings of the National Academy of Sciences* **2023**, *120*, e2300565120.

[3] A. P. Bartók, R. Kondor, G. Csányi, *Physical Review B* **2013**, *87*, DOI 10.1103/PhysRevB.87.184115.

[4] H. Liu, M. M. Smedskjaer, M. Bauchy, *Phys. Rev. B* **2022**, *106*, 214206.

[5] L. Himanen, M. O. J. Jäger, E. V. Morooka, F. Federici Canova, Y. S. Ranawat, D. Z. Gao, P. Rinke, A. S. Foster, *Computer Physics Communications* **2020**, *247*, 106949.

[6] F. H. Stillinger, T. A. Weber, *Physical Review B* **1985**, *31*, 5262.

[7] J. F. Justo, M. Z. Bazant, E. Kaxiras, V. V. Bulatov, S. Yip, *Phys. Rev. B* **1998**, *58*, 2539.
